# ST-segment elevation myocardial infarction in Nail–Patella syndrome with anomalous coronary anatomy and aneurysms: a case report

**DOI:** 10.1093/ehjcr/ytae188

**Published:** 2024-04-18

**Authors:** Kyle Varkoly, Akarsh Parekh, Jason Kaplan, Michael Blair DeYoung

**Affiliations:** Department of Internal Medicine, McLaren Macomb Hospital, 1000 Harrington St, Mount Clemens, MI 48043, USA; Michigan State University College of Human Medicine, 15 Michigan St NE, Grand Rapids, MI 49503, USA; Michigan State University College of Human Medicine, 15 Michigan St NE, Grand Rapids, MI 49503, USA; Department of Cardiovascular Medicine, Ascension Macomb Medical Center, 11800 Twelve Mile Rd, Warren, MI 48093, USA; Michigan State University College of Human Medicine, 15 Michigan St NE, Grand Rapids, MI 49503, USA; Department of Cardiovascular Medicine, McLaren Macomb Hospital, 1030 Harrington St, Mt Clemens, MI 48043, USA; Department of Cardiovascular Medicine, Ascension Macomb Medical Center, 11800 Twelve Mile Rd, Warren, MI 48093, USA; Department of Cardiovascular Medicine, McLaren Macomb Hospital, 1030 Harrington St, Mt Clemens, MI 48043, USA

**Keywords:** Nail–Patella syndrome, Coronary aneurysm, Connective tissue disease, STEMI, PCI, Case report

## Abstract

**Background:**

Nail–Patella syndrome (NPS) is an autosomal-dominant pleiotropic condition characterized by pelvic and skeletal abnormalities and most commonly affecting a tetrad of nails, knees, elbows, and iliac horns, the iliac horns being pathognomonic for the condition. The most well-documented extra-skeletal manifestation is renal involvement with alteration in Type III collagen. No documented cases of NPS with anomalous coronary arteries or aneurysms, acute coronary occlusion, or successfully coronary interventions exist in the medical literature.

**Case summary:**

A 62-year-old female with a medical history significant for NPS diagnosed 50 years ago presented to the emergency department with a chief complaint of chest pain. She recently developed end-stage renal disease managed with peritoneal dialysis within the last year. Angiography revealed 100% right coronary artery occlusion with an anomalous take-off from the left circumflex artery. She demonstrated diffuse coronary aneurysms in the right coronary artery, mid-left anterior descending artery, and other epicardial vessels. Two drug-eluting stents were placed in overlapping fashion. Following careful apposition, the aneurysmal segment was successfully stented without complication. The patient was discharged without complication 2 days later.

**Discussion:**

Our case shows the first reported case of coronary vascular anomalies and successful coronary revascularization in a patient with NPS in the medical literature. Given the recently reported vascular anomalies and known collagen alterations seen in patients with the genetic disorder, clinicians should suspect further systemic vascular anomalies with their own unique therapeutic challenges when encountering patients with this rare genetic syndrome.

**Learning points**

- Nail–Patella syndrome (NPS) is a rare pleiotropic disease not previously appreciated to be an antecedent to coronary vascular anomalies.
- Our patient demonstrated a plethora of rare coronary epicardial anomalies with an anomalous left circumflex (LCx), aneurysmal right coronary artery, aneurysmal mid-left anterior descending artery, aneurysmal proximal circumflex artery arising from the right coronary artery, and aneurysmal right obtuse marginal branch of the anomalous LCx artery during angiography.
- Interventionalists should be aware of possible vascular and epicardial abnormalities when intervening on patients with NPS.

## Introduction

Nail–Patella syndrome (NPS), also known as hereditary onycho-osteodysplasia or Fong disease, is a rare autosomal-dominant pleiotropic condition of pelvic and skeletal abnormalities. It more commonly affects a tetrad of nails, knees, elbows, and iliac horns, with the iliac horns being pathognomonic for the condition. The most well-documented extra-skeletal manifestation of NPS is renal involvement, affecting a rough estimate of 12–55% of patients with NPS.^1^ Nail–Patella syndrome is caused by mutations in the LX1B gene, which is associated with abnormal Type III collagen distribution.^1–4^

## Summary figure

**Table:** 

Day 0: Patient presentation	The patient presented with inferior STEMI with posterior extension on ECG
Day 0: Workup and treatment	The patient was given ACS medications and immediately sent to a cardiac catheterization laboratory
Day 0: Coronary angiography	Angiography revealed 100% RCA occlusion with an anomalous take-off of the LCx. Diffuse coronary aneurysms were demonstrated in the RCA, mid-LAD, proximal Cx artery arising from the RCA, and right obtuse marginal branch of the anomalous Cx artery
Day 0: Intervention #1	RCA was successfully stented over the stenotic lesion. Grade III TIMI Flow was observed immediately after the procedure
Day 0: Intervention #2	An additional DES was placed over the distal RCA segment to approximate the RCA aneurysm in overlapping fashion with the initial DES
Day 0: Intervention #3	Following complete stent placement, our patient experienced hypotension with arrhythmias. Following brief use of an intra-aortic balloon pump (IABP) intraoperatively due to concerns over contributing to cardiogenic shock, the arrythmias and hypotension ceased and IABP was weened
Day 2: Patient discharge	The patient was successfully discharged 2 days later without further complication
Day 15: Outpatient follow-up	Our patient followed up without recurrence of chest pain and was compliant with medical therapy
Day 21: Intervention #4	LCx was revascularized successfully without complication

STEMI, ST-segment elevation myocardial infarction; ECG, electrocardiogram; ACS, acute coronary syndrome; RCA, right coronary artery; LCx, left circumflex artery; LAD, left anterior descending artery; Cx, circumflex; TIMI, thrombolysis in myocardial infarction; DES, drug-eluting stent.

Coronary arterial aneurysms (CAAs) are greater than 1.5-fold local dilatations in coronary arteries. Their rate of incidence in the general population ranges from 0.3 to 5.3% with a mean pooled cohort analysis of 1.65%.^5^ Patients with NPS are presently not known to have inherent CAAs. Genetic studies have yet to identify coronary or vascular anomaly subtypes in patients with NPS.^1,3,4^ Coronary arterial aneurysms have a higher incidence in mixed connective tissue diseases (CTDs) affecting collagen distribution, such as the well-studied Ehlers–Danlos syndrome (EDS) and Marfan syndrome.^5–7^ Both EDS and NPS are a subtype of CTD affecting Type III collagen. Upon autopsy of a female patient with the vascular subtype of EDS who died of a splenic arterial aneurysmal rupture, it was found that the patient additionally had multiple right coronary arterial aneurysms, with no CAAs identified in the left coronary artery.^7^ Coronary aneurysms have also been reported in the left coronary artery in Loeys–Dietz syndrome, a lesser-known mixed CTD.^8^ Furthermore, atypical vascular malformations such as internal carotid artery aplasia have been reported in a patient with NPS.^9^

In this study, we present a case of a patient with NPS who arrived with an ST-segment elevation myocardial infarction (STEMI) and presented with an abnormal and challenging coronary anatomy. After studying the vascular nature of the genetic disorder and after careful stent apposition and deployment, we revascularized our patient without complication.

## Case summary

**Figure 1:**
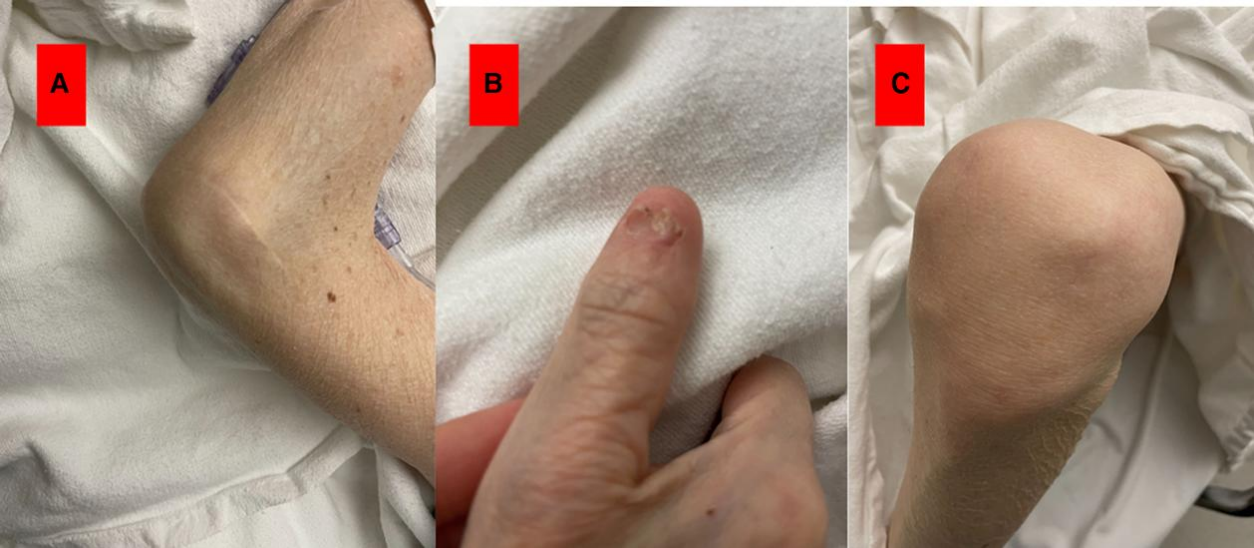
The patient demonstrating phenotypic characteristics common in Nail–Patella syndrome. (*A*) Elbow deformity. (*B*) Fingernail dysplasia. (*C*) Abnormal patella.

**Figure 2:**
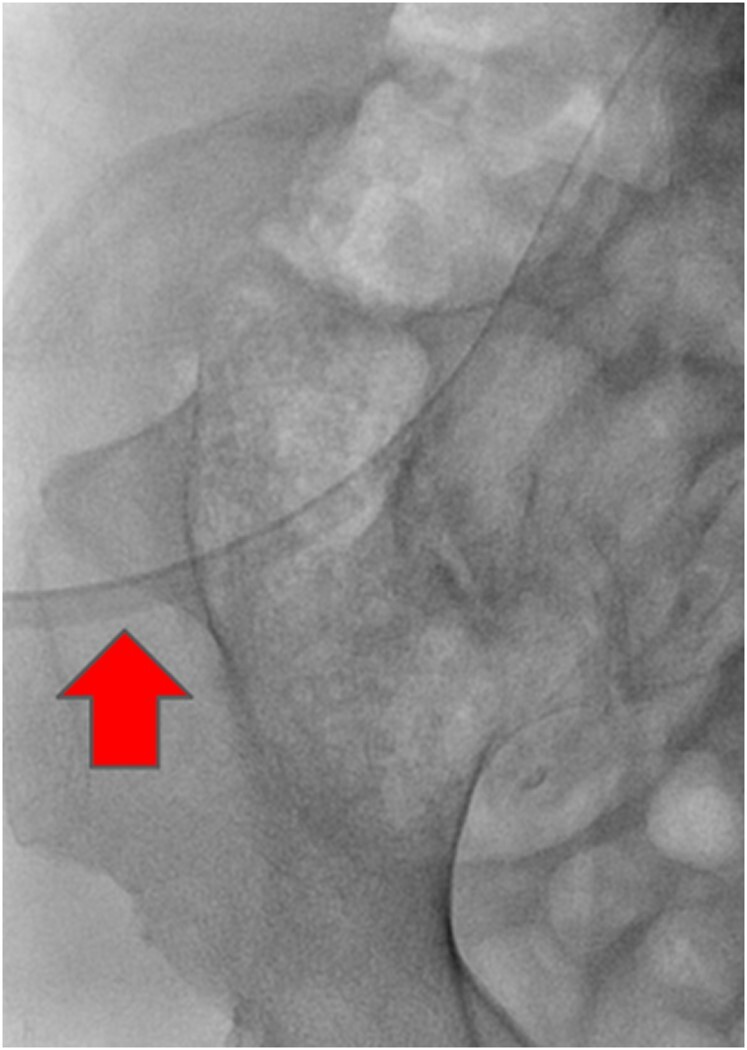
An anteroposterior right pelvic radiograph demonstrating the presence of a triangular osseous protrusion from the posterior aspect of the right ilia, the right iliac horn.

A 62-year-old female presented to the emergency department with a chief complaint of chest pain, nausea, and vomiting. Her medical history included NPS diagnosed at age 12 and end-stage renal disease managed with peritoneal dialysis within the last year. Home medications at the time of presentation included carvedilol 25 mg twice a day, simvastatin 40 mg daily, calcitriol 0.5 mg daily, and sodium bicarbonate 650 mg three times a day. Past operations included a remote history of hysterectomy and peritoneal dialysis placement within the past year. Our patient had no known allergies. Social history included a remote history of cigarette smoking. Family history was unable to be obtained as our patient was an adopted case. Our patient’s known history of NPS was corroborated through characteristic physical examination findings (*Figure 1A–C*). Ultimately, radiographic imaging demonstrating iliac horns, which are pathognomonic for the syndrome, confirmed the diagnosis (*Figure 2*). She had no known history of Kawasaki disease in childhood.

**Figure 3:**
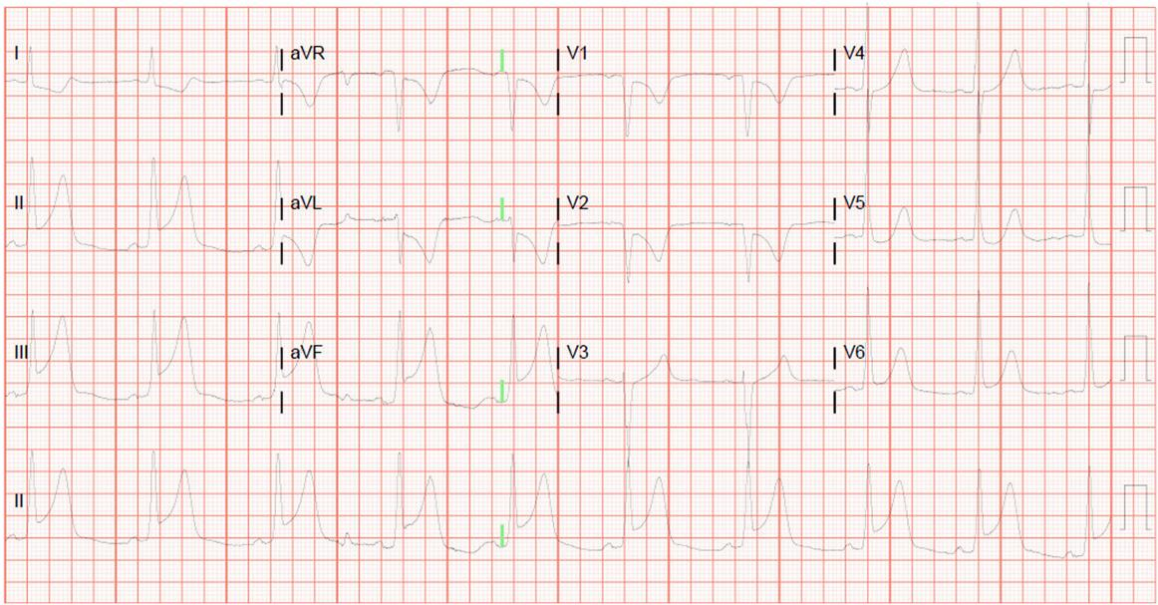
Twelve-lead electrocardiogram concerning for an inferior ST-elevated myocardial infarction with posterior extension.

**Figure 4:**
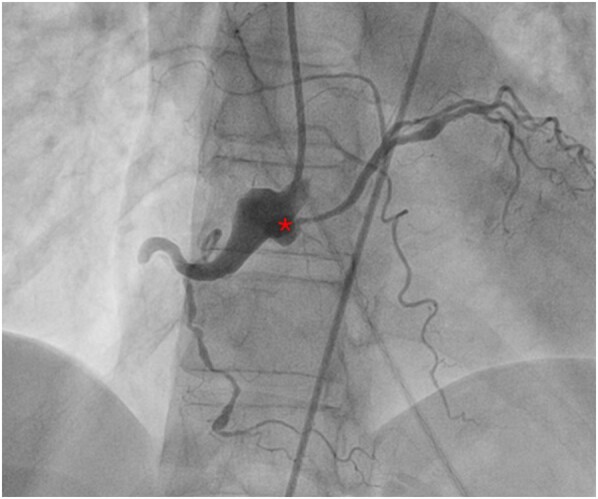
A right coronary angiography demonstrating a 100% occlusion of the proximal right coronary artery as well as an anomalous left circumflex artery off the right coronary ostium. *Approximate origin of the anomalous left circumflex artery.

On arrival, our patient presented with a chief complaint of chest pain. Pertinent positive and negative presenting physical examination findings include diaphoresis and stable vital signs without hypotension. She was found to have an inferior STEMI with posterior extension on initial electrocardiogram (ECG; *Figure 3*). Following this ECG, the differential broadened to spontaneous coronary artery dissection, aortic dissection, pulmonary embolism, pneumothorax, oesophageal rupture, cardiac tamponade, and an inferior infarction. Our patient’s maximum troponin level, the initial troponin level on admission, was 2679.9 ng/L. Brain natriuretic peptide levels (BNP) were elevated at 7595 pg/dL. A STEMI alert was activated, and the patient was emergently taken to the catheterization laboratory for coronary angiography for her probable inferior infarction. Bedside transthoracic echocardiography (TTE) was not performed in the interest of preserving myocardial tissue in the setting of STEMI requiring emergent revascularization. Coronary angiography revealed diffuse coronary aneurysms in the right coronary artery (RCA), a complete acute occlusion of the RCA, and an anomalous left circumflex (LCx) artery off the right coronary ostium (*Figure 4*; Supplementary material online, *Video S1*). A decision was made to intervene on the RCA. Additionally, coronary aneurysms were demonstrated in the RCA, mid-left anterior descending artery, proximal circumflex artery arising from the RCA, and right obtuse marginal branch of the anomalous circumflex artery. The anomalous LCx arising from the right coronary sinus coursed posterior to the aorta. The RCA was deemed to be the culprit lesion.

**Figure 5:**
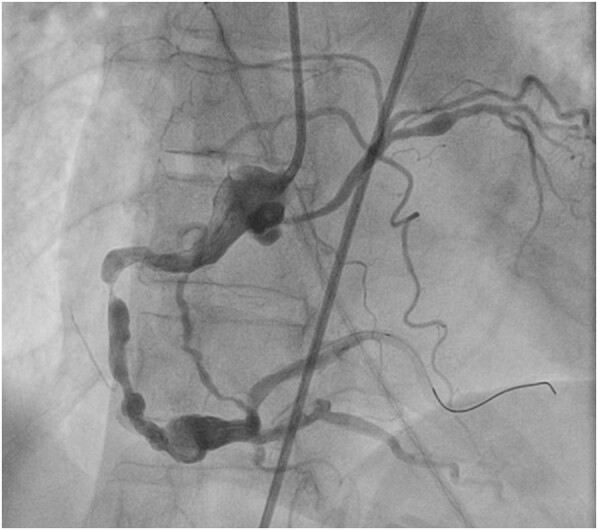
A right coronary angiography after wiring demonstrating an aneurysmal right coronary artery.

**Figure 6:**
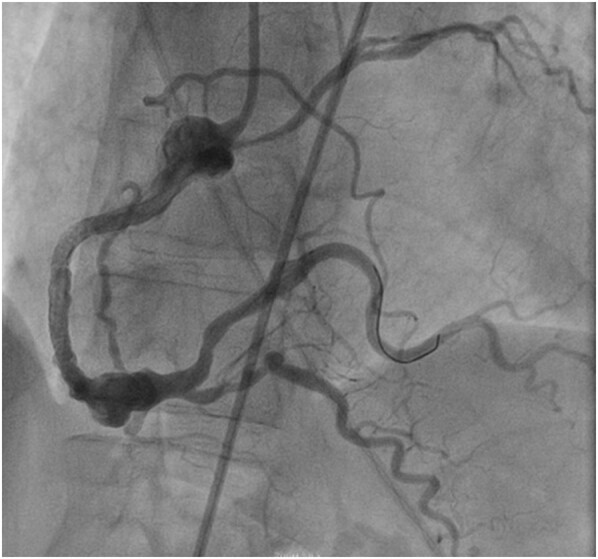
A right coronary angiography demonstrating two 4.0 mm overlapping stents placed in the proximal-to-mid right coronary artery.

The aneurysmal appearance throughout the mid-to-proximal segments of the RCA created a challenge for proper stent apposition (*Figure 5*). Two 4.0 × 4.0 mm drug-eluting stents were placed in the proximal-to-mid RCA in an overlapping fashion, post-dilated with a 4.0 mm non-compliant balloon (*Figure 6*; Supplementary material online, *Video S1*). Our patient did require brief intraoperative use of an intra-aortic balloon pump for transient reduced cardiac output due to reperfusion arrhythmias contributing to hypotension and cardiogenic shock. She was started on a dual antiplatelet therapy of acetylsalicylic acid 81 mg daily and ticagrelor 90 mg twice a day. She was discharged home 2 days following intervention without complication. She continued her follow-up in the outpatient setting for revascularization of her left circumflex, which was completed successfully on another planned hospitalization. Following the above interventions and proper outpatient follow-up, our patient is now on clopidogrel 75 mg q.d., which she will take indefinitely. A follow-up TTE following her left circumflex intervention revealed no regional wall motion abnormalities.

## Discussion

Nail–Patella syndrome is a known autosomal-dominant pleiotropic disorder with concerns for vascular pathologies due to its effects on Type III collagen, particularly if renal involvement is present. Much like the nails, skeletal bones, and tendons, the vascular endothelial walls are made of collagen, particularly Types I and III. In pathologic skeletal conditions, there is an abnormally high collagen Type III/Type I ratio, as Type III collagen is known to have less tensile strength when compared with Type I collagen.^2^ Nail–Patella syndrome is known to lead to an irregular distribution of Type III collagen, particularly in phenotypes with renal involvement.^3^

Coronary arterial aneurysms are most commonly caused by atherosclerosis. Coronary arterial aneurysms have a higher rate of incidence in atherosclerotic males than in atherosclerotic females (2.2 vs. 0.5% at a single-centre study).^5^ Hereditary CTD, such as EDS and Marfan syndrome, have been shown to cause CAAs, driven by a gene mutation with resultant transforming growth factor-β (TGF-β) overactivity causing cystic medial necrosis.^5,6^ However, cystic medial necrosis has also been identified in patients with CAAs without Marfan syndrome, and in these patients, medial necrosis, fibrosis, and atherosclerosis also directly correlated with age.^6^

Type III collagen is implicated in the vascular subtype of EDS. Upon autopsy of a female patient with EDS who died of splenic arterial aneurysmal rupture, it was found that the patient was uncovered to have a right coronary aneurysm as well.^7^ Loeys–Dietz syndrome, another CTD affecting Type III collagen, has also been reported to have coronary aneurysms.^8^ Already, hereditary internal carotid artery aplasia has been reported as a vascular abnormality in a patient with NPS.^9^ Given the increased identification of anomalous vascular abnormalities in patients with CTDs, it is reasonable to identify other potential drivers in NPS potentially accelerating CAA formation.

An anomalous LCx take-off of the right coronary sinus is classified as a coronary artery anomaly, which are anomalies identified in <1% of the general population.^10^ The anomalous course usually continues posterior to the aorta and is considered benign. The rate of incidence of an anomalous LCx is overall rare, which approached 0.26% in a single-centre retrospective observational study of 2684 coronary angiography procedures, which was corroborated through other observational coronary angiography studies.^11,12^ Identification of this anomalous coronary artery is important to avoid selective atherosclerosis of this or nearby arteries due to nearby mechanical forces that could possibly invoke spontaneous coronary artery dissection. Such an anomalous take was identified in our patient.

No studies have formally examined NPS and its effects on vascular or coronary malformations. Additionally, no gene mutations have been identified as drivers of vascular or coronary malformations in patients with NPS.^1,3,4^ Due to its known effects on collagen, it is reasonable for interventionists to have concerns for vascular pathology in NPS. Although rare, when managing patients with NPS, physicians should now be aware of a potentially complex anatomy, making routine procedures extensively more difficult, as seen in our patient. Given our limited insights into the vasculature of these patients, additional recommendations for the management of these patients include a full body computed tomography with brain to pelvis imaging, substantially improving outcomes in patients with known aneurysmal non-atherosclerotic vascular disease.^13^

Our patient case shows a rare anatomical variant of coronary aneurysm and anomalous take of LCx, and is the first reported case showing coronary aneurysms in a patient with NPS in the medical literature, a potential association not appreciated in prior genetic reviews of the syndrome.^1,3,4^ Our patient with NPS had a rare coronary anomaly of coronary aneurysm, presenting with a complete occlusion, and was successfully revascularized without intra-hospital complication following additional stent apposition and deployment. No formal guidelines from the European Society of Cardiology exist for revascularization strategies in patients with CTD.^14^ However, time is of the essence in preserving myocardial tissue in the context of STEMI.^15^ Thus, initial revascularization in the case of our patient was reasonable. Ideally, intravascular ultrasound would have been employed prior to stent deployment. However, given the patient’s haemodynamic instability, this was not feasible.

## Conclusion

This is the first reported case of coronary vascular anomalies seen in a patient with NPS and is the first successful coronary revascularization in a patient with NPS in the medical literature. Given the potential vascular abnormalities and need for cardiac intervention in patients with NPS, it is reasonable for future studies to examine vascular malformations in this patient population.

## Supplementary Material

ytae188_Supplementary_Data

## Data Availability

The data underlying this article will be shared on reasonable request to the corresponding author.

## Lead author biography

Dr Kyle Varkoly is in training for internal medicine at McLaren Macomb Hospital in Michigan. Introduced to academic cardiology at the University of Florida with his first co-authorship investigating the relationship between markers of inflammation in periodontal infections and atherosclerosis, his academic interests have continued to bloom with his latest papers identifying RNA viral gene signatures in patients developing cardiomyopathy following chemotherapy and articles on viral-derived thrombolytics. His areas of interest include coronary angiography, intervention, and reducing overall cardiovascular mortality. He is continuing his training at his home institution’s flagship hospital, McLaren-Flint, for cardiovascular medicine starting July 2024.

## Supplementary material

**Consent:** The authors confirm that written consent for submission and publication of this case report, including the images and associated text, has been obtained from the patient in line with COPE guidance.

**Funding:** None declared.
